# Nova1 or Bim Deficiency in Pancreatic β-Cells Does Not Alter Multiple Low-Dose Streptozotocin-Induced Diabetes and Diet-Induced Obesity in Mice

**DOI:** 10.3390/nu14183866

**Published:** 2022-09-18

**Authors:** Manoja K. Brahma, Peng Xiao, Madalina Popa, Javier Negueruela, Valerie Vandenbempt, Stéphane Demine, Alessandra K. Cardozo, Esteban N. Gurzov

**Affiliations:** 1 Signal Transduction and Metabolism Laboratory, Laboratoire de Gastroentérologie Expérimental et Endotools, Université Libre de Bruxelles, 1070 Brussels, Belgium; 2 Inflammatory and Cell Death Signaling in Diabetes, Laboratoire de Gastroentérologie Expérimental et Endotools, Université Libre de Bruxelles, 1070 Brussels, Belgium

**Keywords:** pancreatic β-cells, Nova1, Bim, obesity, diabetes

## Abstract

The loss of functional pancreatic β-cell mass is an important hallmark of both type 1 and type 2 diabetes. The RNA-binding protein NOVA1 is expressed in human and rodent pancreatic β-cells. Previous in vitro studies indicated that NOVA1 is necessary for glucose-stimulated insulin secretion and its deficiency-enhanced cytokine-induced apoptosis. Moreover, Bim, a proapoptotic protein, is differentially spliced and potentiates apoptosis in NOVA1-deficient β-cells in culture. We generated two novel mouse models by Cre-Lox technology lacking Nova1 (βNova1^−/−^) or Bim (βBim^−/−^) in β-cells. To test the impact of Nova1 or Bim deletion on β-cell function, mice were subjected to multiple low-dose streptozotocin (MLD-STZ)-induced diabetes or high-fat diet-induced insulin resistance. β-cell-specific Nova1 or Bim deficiency failed to affect diabetes development in response to MLD-STZ-induced β-cell dysfunction and death evidenced by unaltered blood glucose levels and pancreatic insulin content. In addition, body composition, glucose and insulin tolerance test, and pancreatic insulin content were indistinguishable between control and βNova1^−/−^ or βBim^−/−^ mice on a high fat diet. Thus, Nova1 or Bim deletion in β-cells does not impact on glucose homeostasis or diabetes development in mice. Together, these data argue against an in vivo role for the Nova1-Bim axis in β-cells.

## 1. Introduction

Humans and vertebrates respond to circulating glucose levels by secreting insulin from β-cells in the pancreas. The loss of functional β-cell mass is an important hallmark of type 1 diabetes (T1D) and can contribute to the development of type 2 diabetes (T2D). This decline in β-cell mass is associated with apoptosis [1,2]. Genome Wide Association Studies (GWAS) allowed the understanding of transcriptional regulation and identified several candidate genes linked to β-cell dysfunction in T1D and T2D [3]. However, the gene networks contributing to dysregulated insulin secretion and the pathogenesis of diabetes is not fully understood. During transcription, the cellular machinery processes pre-mRNAs through a complex series of events before a mature mRNA is formed. Alternative mRNA splicing is one of the important post-transcriptional mechanisms in which a single gene produces multiple mRNA transcripts that generate different protein isoforms [4]. Abnormal alternative splicing has been associated with many diseases in the adipose tissue, liver, and brain [5,6,7]. In the pancreas, alternative splicing in β-cells has recently gained momentum with multiple lines of evidence suggesting its association with β-cell dysfunction in both T1D and T2D [8,9,10,11,12].

Alternative splicing of mRNA is achieved through multiple RNA-binding proteins (RBPs) that bind to pre-mRNAs to facilitate intron removal by defining the exon slice recognition site. Impaired RBP function has been linked to diabetes development [13]. RBPs are enriched and play an important role in β-cell function and survival [8,14]. Interestingly, RNA-Sequencing (RNA-Seq) of human tissues has revealed that pancreatic islets express several neuron-specific splicing factors [15], indicating that (i) β-cells and neurons share similar splicing signature and factors and (ii) there is a crucial role of neuron-enriched splicing factors present in β-cell function [16]. The Neuro-Oncological Ventral Antigens (NOVA) are a family of two RBPs (NOVA1 and NOVA2) that are associated with approximately 700 alternative splicing events in neurons, both in the cytoplasm and nucleus [17]. By using a combination of RNA-Seq, siRNA technology, and functional studies, Villate O et al. showed that Nova1 is necessary for glucose-stimulated insulin secretion in rodent INS-1E β-cells. Furthermore, the silencing of NOVA1 in rodent β-cells and dispersed human islets induces both basal and cytokine-induced apoptosis [18]. Moreover, the authors demonstrated that Bcl-2 Interacting Mediator of cell death (Bim), a proapoptotic protein [19], was differentially spliced and potentiated apoptosis in Nova1-deficient β-cells. In addition, Bim has been shown to modulate several apoptotic events in β-cells under stress [2,19,20,21,22]. While results from this in vitro finding strongly suggest a crucial activity of NOVA1/BIM in β-cell function and survival, the role of NOVA1 in β-cell functioning in vivo remains elusive. Interestingly, a recent finding by Vernia S et al. showed that adipocyte-specific Nova1-deficient mice exhibit augmented thermogenesis and improved glycemia, suggesting an important physiological metabolic role of Nova1 in vivo [23]. Collectively, based on these findings, we hypothesized that β-cell-specific deletion of Nova1-Bim will have a major impact on β-cell function and survival in vivo. To address this, we used Cre-recombinase technology to delete Nova1 or Bim specifically in β-cells in mice and characterized the impact of deletion of the genes on glucose homeostasis in vivo.

## 2. Materials and Methods

### 2.1. Animal Models and In Vivo Procedures

Mice were studied in accordance with the ethics protocol approved by the Commision d’Ethicque du Bien-Être Animal (CEBEA), Faculté de Médecine, Université libre de Bruxelles (dossier No. 732). β-cell-specific suppression of Nova1 or Bim were generated using Cre-LoxP technology on C57BL/6N background. Homozygous Nova1floxed mice (Nova1^fl/fl^ Genome way, Lyon, France) were generated by insertion of floxed sequence in addition to the endogenous exons 1–3 of Nova1 in chromosome 12 and kindly provided by the ULB Center for Diabetes Research (Brussels, Belgium). Nova1^fl/fl^ mice were crossed with mice harboring one allele of Cre-recombinase under the mouse Ins1 promoter (Ins1Cre; Jackson Laboratory, Bar Harbor, ME, USA) to generate mice heterozygous for the floxed Nova1 gene and expressed the Ins1Cre transgene (Nova1^+/fl/Cre^). Nova1^+/fl/Cre^ mice were crossed with Nova1^fl/fl^ mice to generate β-cell-specific Nova1 knockout mice (βNova1^−/−^). β-cell-specific Bim knockout mice (βBim^−/−^) were generated by using a similar approach. Thus, Bim^fl/fl^ mice [24,25] (kindly provided by Philippe Bouillet, The Walter and Eliza Hall Institute, Melbourne, Australia) were crossed with Ins1Cre to generate βBim^−/−^ mice. Animals were housed at 22 °C on a 12:12-h light-dark cycle with ad libitum access to food and water. We have used littermates for our study.

### 2.2. Diabetes Induction and High Fat Diet Treatment

To induce diabetes in mice, we utilized a mouse multiple low-dose streptozotocin (MLD-STZ) mouse model. Male mice were injected with streptozotocin (45 mg/kg body weight) prepared in a 0.1 M citrate buffer (pH 4.5) for 5 consecutive days. Body weight and blood glucose (blood drawn from tail vein) were measured every week. Body composition was measured before STZ injection and every 2 weeks. To further test the impact of the loss of Nova1 or Bim, we used a second mouse model of β-cell stress, where we fed mice with a high fat diet (HFD, 60% kcal fat, D16042106i Research Diets, New Brunswick, NJ, USA) for 12 weeks. Tissues were collected under non-fasted conditions. Body fat, lean mass, and total water mass were determined by using EchoMRI^TM^3-in-1 (Houston, TX, USA). We have used the following cohorts for the experiments: 11–12-week-old male Nova1^fl/fl^ and βNova1^−/−^ for MLD-STZ; 10–11-week-old male Nova1^fl/fl^ and βNova1^−/−^ for high fat diet; 9–10-week-old male Bim^fl/fl^ and βBim^−/−^ for MLD-STZ; 10–11-week-old male Bim^fl/fl^ and βBim^−/−^ for high fat diet.

### 2.3. Isolation of Mouse Islet and Fluorescence-Activated Cell Sorting (FACS) of β-Cells

Mouse islets were isolated by digesting the pancreas with collagenase (Sigma-Aldrich, St Louis, MI, USA; cat. #C9263) prepared in serum-free M199 media (Gibco-Thermo Fisher, Waltham, MA, USA; cat #22350) at a concentration of 0.8 mg/mL. Isolated islets were hand-picked under a stereomicroscope two or three times, until a population of pure islets was obtained. 

For FACS-sorting of β-cells, islets were first washed with cell dissociation medium (124 mM NaCl, 5.4 mM KCl, 0.8 mM MgSO_4_, 1 mM Na_2_PO_4_, and 10 mM HEPES). Following the washing, pancreatic islets were disaggregated by gentle continuous pipetting in trypsin (1 mg/mL; Sigma-Aldrich; cat. #T9935) and DNase I (Sigma-Aldrich; cat. #10104159001; 1 mg/mL) in a water bath at 37 °C. Dispersed islet cells were centrifuged, and the cell pellet was resuspended with FACS buffer, filtered through 35 µm nylon mesh cell strainer-caped tubes (Fisher Scientific, Hampton, NH, USA; cat. #10100151), and β-cells were purified by light scatter activity and autofluorescence by FACS (BD FACSAria™ III, BD Biosciences, Franklin Lakes, NJ, USA) as previously described [26,27]. After sorting, β-cell fraction was collected in a tube containing RPMI 1640 (Gibco-Thermo Fisher, cat. #61870044). Cells were centrifuged and the pellet was lysed using RIPA buffer and processed for immunoblotting.

### 2.4. Glucose Stimulated Insulin Secretion 

Isolated islets were allowed to recover for 1–2 h at 37 °C (5% CO_2_) in M199 medium supplemented with streptomycin, penicillin, and 10% FBS. Following incubation, groups of ten size-matched islets were incubated with 1 mL of Krebs buffer containing 1.6 mM glucose at 37 °C in 5% CO_2_ incubator for 30 min. For stimulation, islets were switched to a solution of the KRB buffer containing either 2.8 mM (control) or 16.7 mM (stimulated) glucose for 60 min at 37 °C. The supernatant fraction was assayed for secreted insulin by ELISA (Crystal Chem Cat 90080, Crystal Chem Inc., IL, USA) and the islets were collected for measurements of protein for normalization.

### 2.5. Western Blotting

Total protein lysates were isolated in a RIPA buffer (150 mM NaCl, 1 mM EDTA, 50 mM Tris/HCl, 0.5% SDS, 1% NP-40), plus a Halt^TM^ protease and phosphatase inhibitor cocktail (Thermo Fisher, cat #78442). Protein was quantified by a BCA protein assay kit (Thermo Fisher; cat. #PI23227). 50–100 μg protein lysate was separated on polyacrylamide gels and transferred to a 0.22 µM nitrocellulose membrane (Bio-Rad, Hercules, CA, USA). Membranes were immunoblotted with primary antibodies for NOVA1 (MilliporeSigma, Burlington, MA, USA; cat. #07-637), NOVA2 (Proteintech, Rosemont, IL, USA; cat. #55002-1-AP), BIM (Cell Signaling Technology Inc., Danvers, MA, USA; cat. #2933, clone C34C5), or GAPDH (Trevigen, Wiesbaden, Germany; cat. #2275-PC-100) in a milk blocking buffer. Proteins were detected using goat anti-rabbit IgG (Dako Agilent, Santa Clara, CA, USA; cat. #P0448) secondary antibody labelled with HRP followed by signal visualization western blot imaging system (Amersham ImageQuant 800 western blot imaging system, Cytiva Life Science, Marlborough, MA, USA).

### 2.6. Histological Studies and Immunohistochemical Staining

The pancreata were harvested from the sacrificed mice after dissection and were washed with saline. The specimens were fixed in 10% buffered formalin (pH 7.4) and embedded into paraffin blocks. The blocks were cut into (7 µm) paraffin sections by a rotator microtome. The sections were stained with Hematoxylin and Eosin (H&E) and with Masson trichrome stains as indicated [28,29].

7 µm thick paraffin sections were mounted on positively charged slides and subjected to immunohistochemical procedures. Paraffin sections were dewaxed, and antigen unmasking was performed using a heated citrate buffer (10 mM). Sections were permeabilized using triton (0.1%) followed by blocking with 2% milk (15 min) and 10% normal goat serum (30 min room temperature) was used to block non-specific binding sites. Specimens were incubated with primary antibodies overnight (Insulin, Dako Agilent; cat. #A0564; Glucagon, Sigma-Aldrich; cat. #G2654; CD68, Cell Signaling; cat. #97778) at 4 °C followed by 1 h incubation with the secondary antibody conjugated to the fluorochrome for insulin (Goat anti-Guinea Pig IgG Thermo Fisher, cat. #A21435 or #A11073), glucagon (Donkey anti-Mouse IgG, Sigma-Aldrich, cat. #A21202 or #A32794), and CD68 (Donkey anti-Rabbit IgG, Sigma-Aldrich, cat. #A31571). Specimen slices incubated only with secondary antibody were used as negative controls. Nuclei were counterstained with DAPI (Vector Laboratories, Burlingarme, CA, USA; cat. #H-1200) before mounting. Images were analyzed on a fluorescent microscope (Axio Observer D1, Carl Zeiss, Oberkochen, Germany). The quantification of the percentage of insulin and glucagon positive cells per islet was performed within the islets, which were analyzed using Cell profiler automated counting software (Broad Institute, Cambridge, MA, USA). 

### 2.7. Pancreatic Insulin Content

Pancreas pieces close to the intestine and liver were collected followed by homogenization with ethanol/water/acid (75/23.5/1.5, % *v/v*). Homogenized samples were incubated on a tube rotator for 24 h at 4 °C and centrifuged at 1500 g for 30 min. Supernatant was collected and used for insulin measurement using a commercial Insulin ELISA kit (Crystal Chem Inc., Chicago, IL, USA; cat. #90080). Insulin content was expressed as µg insulin/g pancreas weight (µg/g). 

### 2.8. Intraperitoneal Glucose and Insulin Tolerance Tests (IPGTT and ITT)

An intraperitoneal glucose tolerance test (IPGTT) was carried out after a 6 h fast in age-matched male mice, and 2 g/Kg glucose/body weight was administered by intraperitoneal injection. For MLD-STZ studies, 1 g/Kg body weight of glucose was administered. For the insulin tolerance test (ITT), regular human insulin (Sigma-Aldrich; cat. #I9278; 0.65 U/kg in saline) was administered intraperitoneally to 4 h fasted mice. Blood was collected from the tail vein before (basal, 0 min) and at 15, 30, 60, 90, and 120 min after glucose or insulin administration using a commercial glucometer (Accu-Check Perfoma, Roche, Basel, Switzerland).

### 2.9. Statistical Analysis

Analyses were performed with GraphPad Prism 8.4 software (Prism, San Diego, CA, USA). All the results are expressed as mean ± SEM. Student’s t-tests or, where appropriate, two-way analysis of variance (ANOVA) was performed. Statistical analysis was performed using the criterion for significance of *p* < 0.05 for all comparisons.

## 3. Results

### 3.1. Generation of Mice with β-Cell-Specific Loss of Nova1 (βNova1^−/−^) or Bim (βBim^−/−^) 

**Figure 1:**
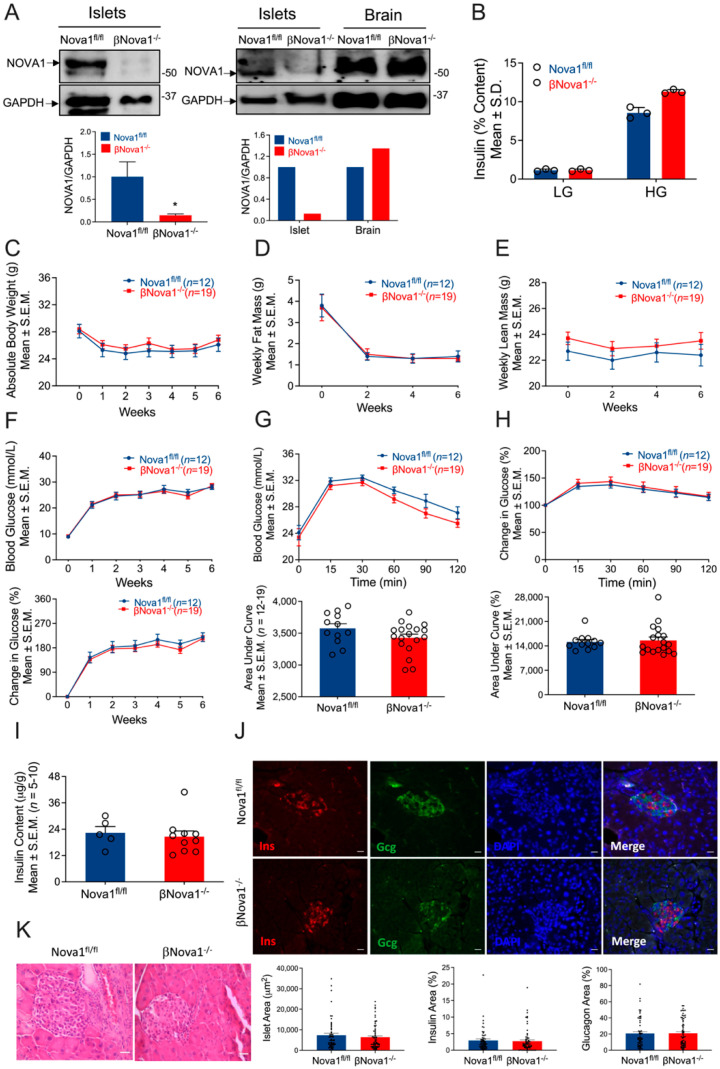
Impact of β-cell-specific Nova1 deletion on glucose homeostasis. Nova1 was deleted in β-cells of mice using Cre-LoxP technology. Nova1 deletion was confirmed in isolated islets from Nova1^fl/fl^ or βNova1^−/−^ mice by immunoblotting using anti-NOVA1 antibody (**A**). Ex vivo glucose stimulated insulin secretion was performed in isolation from Nova1^fl/fl^ or βNova1^−/−^ mice in the presence of low or high glucose (**B**). To test the impact of diabetes on glucose homeostasis Nova1^fl/fl^ or βNova1^−/−^ mice were injected with 5-days of MLD-STZ and followed for 6 weeks. Body weight (**C**), body composition (**D**,**E**), and blood glucose (**F**; **top and bottom panel**) were monitored following STZ injection. GTT was performed in 6 h fasted mice after 6 weeks of STZ administration. Blood glucose was measured at indicated time points and expressed as mmol/L (**G**) or % change in blood glucose normalized to time 0 (**H**). Figure **G** (**bottom panel**) and **H** (**bottom panel**) represent glucose areas under the curve. Pancreatic insulin content was measured in acid–ethanol extracted fraction using a commercial ELISA kit (**I**). Pancreatic sections were stained for insulin, glucagon, and DNA (**J**). Scale bar, 20 µm. Representative of 3 mice per genotype (**J**). **Bottom panel** (**J**) showing islet area, insulin, and glucagon positive cells (calculated as % of total islet area). Histology of mouse pancreas stained by Hematoxylin and Eosin (**K**). Destruction and distortion of endocrine cells in STZ-diabetic mice with the presence of dead and inflammatory cells. Scale bar, 25 µm. Data are presented as Mean ± SEM. * *p* < 0.05 vs. Nova1^fl/fl^ mice.

To directly investigate the effects of β-cell-specific Nova1 knockout in mice, we used the Cre-LoxP recombination system. Briefly, Nova1^fl/fl^ mice were crossed with transgenic mice expressing the Cre recombinase under the control of the mouse insulin promoter (Ins1Cre). Nova1 deletion was confirmed by PCR analysis of genomic DNA isolated from ear notches (data not shown) and immunoblot analysis of Nova1 protein expression in isolated islets. As shown in Figure 1A (left panel and right panel), immunoblot analysis confirmed a robust deletion (85%) of Nova1 in isolated islets. Since Nova1 is a neuron-specific alternative splicing factor, we confirmed that Nova1 deletion was specific to β-cells and its expression not altered in the brain (Figure 1A). In addition, Nova2 expression in pancreatic islets was not affected by Nova1 deletion in β-cells (Figure S1).

### 3.2. In Vivo Glucose Homeostasis Was Unchanged in βNova1^−/−^ Mice in Response to MLD-STZ-Induced Diabetes 

To test the impact of Nova1 deletion on β-cells, we first performed in vitro glucose stimulated insulin secretion in the isolated islets from βNova1^−/−^ mice. While basal insulin secretion was unchanged between 2 genotypes, a trend towards increase (+32%) in glucose-stimulated insulin secretion was observed in βNova1^−/−^ mice compared to Nova1^fl/fl^ mice when stimulated with high glucose (Figure 1B). Next, to test our hypothesis that Nova1 deletion exacerbates the impaired glucose tolerance in diabetes, we subjected βNova1^−/−^ and littermate control mice to a model of inflammatory-mediated diabetes by injecting MLD-STZ and followed the glycemia, body composition, and body weight. Nova1 deletion in β-cells did not alter body weight (Figure 1C) or composition (Figure 1D,E and Figure S2A–H). Similarly, blood glucose measured every week was similar in both genotypes (Figure 1F; top and bottom panels) and the mice had no differences in elevated blood glucose (Nova1^fl/fl^: 26 ± 1.1 mmol/L, βNova1^−/−^: 24.6 ± 1.1 mmol/L). At the end of 6 weeks, IPGTT performed in βNova1^−/−^ and littermate controls revealed no change between genotypes in glucose tolerance indicated by blood glucose levels (Figure 1G; top and bottom panels) and % change in blood glucose (Figure 1H; top and bottom panels) after glucose injection. No change in insulin content in the pancreas was observed in these mice (Figure 1I). Finally, we performed immunofluorescent staining with the pancreas sections of the mice to analyze both insulin-secreting β-cells and glucagon-secreting α-cells. Most islets were disrupted in both genotypes and no difference in the islet area or insulin area, or in the glucagon area or macrophage numbers, were observed (Figure 1J and Figure S3A,B). Similarly, histology of pancreas stained by H&E showed no difference between two genotypes (Figure 1K). There are several caveats and concerns about β-cell specific Cre mouse lines, and results should be carefully interpreted with these models [30,31,32]. Importantly, the Ins1Cre mouse model used in the present study does not affect body weights and glucose homeostasis when treated with MLD-STZ compared to C57BL/6N control mice (Supplementary Figure S4A–F) [32]. In addition, we confirmed that the Cre transgene alone does not affect body composition (Supplementary Figure S5A–H). Collectively, these results show that Nova1 deletion in β-cells does not impair glucose homeostasis. 

### 3.3. In Vivo Glucose Homeostasis Was Unchanged in βNova1^−/−^ Mice un Response to High-Fat Diet 

**Figure 2:**
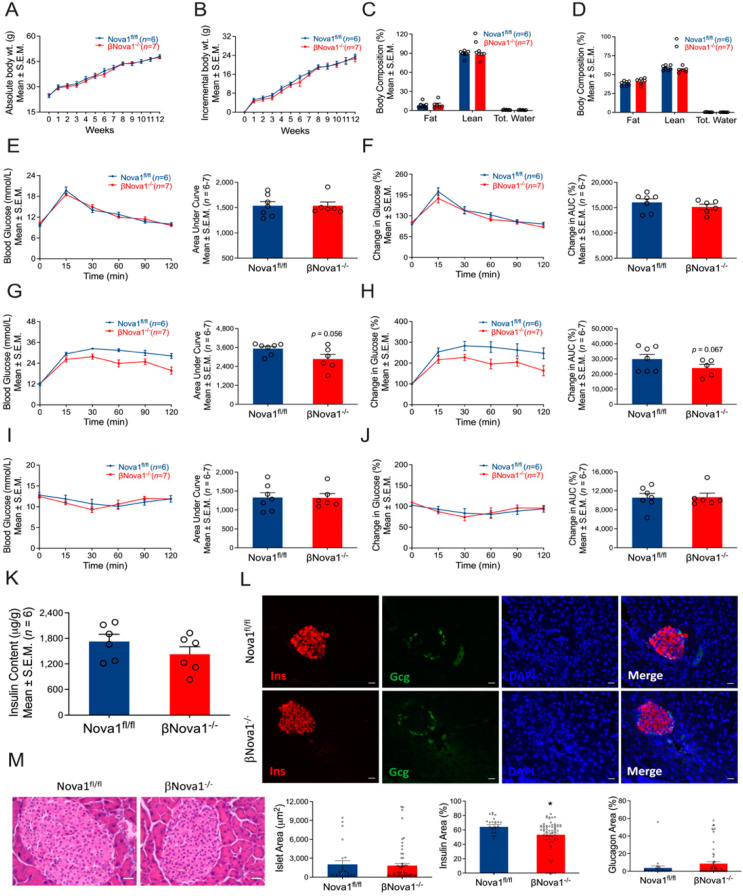
Effect of high-fat diet on glucose homeostasis of βNova1^−/−^ mice. βNova1^−/−^ mice were fed a high-fat diet for 12 weeks. Body weight was measured weekly (**A**,**B**). Incremental body weight (**B**) was calculated as cumulative gain in body weight (g) every week. Body composition (fat mass, lean mass, and total water) was measured before and after 12 weeks of high-fat diet feeding (**C**,**D**). A glucose tolerance test performed before high-fat diet feeding did not show any change in βNova1^−/−^ mice compared to Nova1^fl/fl^ mice (**E**,**F**). After 12 weeks following the high-fat diet, βNova1^−/−^ mice exhibited a trend towards improved glucose tolerance compared to Nova1^fl/fl^ mice (**G**,**H**). However, an insulin tolerance test measured one week after the IPGTT did not show any difference between the two genotypes (**I**,**J**). Pancreatic insulin content was measured in an acid–ethanol extracted fraction using a commercial ELISA kit (**K**). Immunofluorescence staining of pancreatic sections (insulin, glucagon, and DNA) from both high-fat-fed and control Nova1^fl/fl^ and βNova1^−/−^ mice (**L**). Insulin (red staining) is contained in the core zone of the islet, while glucagon (green staining) is found in the mantle zone. Scale bar, 20 µm. Representative of 3 mice per genotype (**L**). **Bottom panel** (**L**) showing islet area, insulin, and glucagon positive cells (calculated as % of total islet area). Histology of mouse pancreas stained by Hematoxylin and Eosin (**M**). Scale bar, 25 µm. Data are presented as Mean ± SEM. * *p* < 0.05 vs. Nova1^fl/fl^ mice.

To further understand the impact of Nova1 deletion in a model of moderate β-cell stress, we induced metabolic stress (obesity and insulin resistance) by feeding the mice a high-fat diet for 12 weeks [25,33]. The high-fat diet (HFD) increased body weight (Figure 2A,B) and fat mass in both βNova1^−/−^ and control mice (Figure 2C,D) after 12 weeks. Before starting the HFD, the glucose tolerance test was not different between βNova1^−/−^ and Nova1^fl/fl^ control mice (Figure 2E,F). After 12 weeks of the HFD, fasting blood glucose was elevated in both Nova1^fl/fl^ (Before HFD: 9.6 ± 0.3, after HFD: 11.9 ± 0.8) and βNova1^−/−^ mice (Before HFD: 10.2 ± 0.3, after HFD: 12.1 ± 0.3). Interestingly, βNova1^−/−^ mice demonstrated a moderate improvement in glucose tolerance at 60 and 120 min after glucose injection. However, the glucose area under curve demonstrated the only trend towards reduction in (*p* = 0.056) in βNova1^−/−^ mice (Figure 2G,H). To assess insulin action, an intraperitoneal insulin tolerance test was performed 1 week after the IPGTT. No change in ITT was observed between the 2 groups (Figure 2I,J). Moreover, pancreatic insulin content was also indistinguishable between Nova1^fl/fl^ and βNova1^−/−^ mice (Figure 2K). To further determine if Nova1 deletion altered insulin expression at the histological level, we investigated the histology of the pancreas via H&E and immunofluorescence analysis on pancreatic sections from high-fat fed Nova1^fl/fl^ and βNova1^−/−^ mice. Interestingly, as depicted in Figure 2L (bottom panel), pancreatic sections from βNova1^−/−^ mice showed a significant reduction in the percentage insulin area (*p* = 0.0163) despite the unchanged islet area between Nova1^fl/fl^ and βNova1^−/−^ mice. However, the H&E staining did not show any notable change in the islet morphology between the two genotypes (Figure 2M). This suggests that although Nova1 deletion in β-cells reduces insulin positive cells, it is not sufficient to bring change in the overall glucose homeostasis at the whole-body level.

### 3.4. Bim Deletion in β-Cells Did Not Alter In Vivo Glucose Homeostasis in Response to MLD-STZ-Induced T1D or High-Fat Diet-Induced Obesity

**Figure 3:**
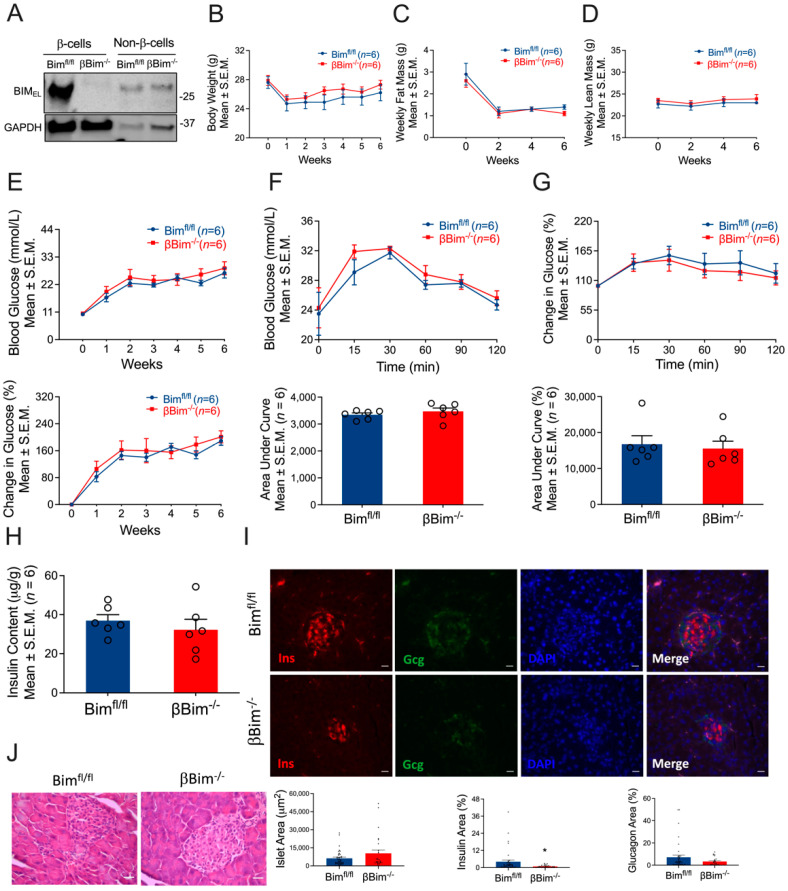
Impact of β-cell-specific Bim deletion on glucose homeostasis. Bim was deleted in β-cells of mice using Cre-LoxP technology. Bim deletion was confirmed in FACS-purified β-cells isolated from islets of Bim^fl/fl^ or βBim^−/−^ mice by immunoblotting using anti-Bim antibody (**A**). To test the impact of diabetes on glucose homeostasis, Bim^fl/fl^ or βBim^−/−^ mice were injected with 5-days of MLD-STZ and followed for 6 weeks. Body weight (**B**), body composition (**C**,**D**), and blood glucose (**E**; top and bottom panel) were monitored following STZ injection. GTT was performed in 6 h fasted mice after 6 weeks of STZ administration. Blood glucose was measured at indicated time points and expressed as mmol/L (**F**) or % change in blood glucose normalized to time 0 (**G**). Figure (**F**) (**bottom panel**) and **G** (**bottom panel**) represent glucose areas under the curve. Pancreatic insulin content was measured in an acid–ethanol extracted fraction using a commercial ELISA kit (**H**). Destruction and distortion of endocrine cells was observed in STZ-diabetic mice with the presence of dead and inflammatory cells. Pancreatic sections were stained for insulin and glucagon and DNA. Scale bar, 20 µm. Representative of 3 mice per genotype (**I**). **Bottom panel** (**I**) showing islet area, insulin, and glucagon positive cells (calculated as % of total islet area). Histology of mouse pancreas stained by Hematoxylin and Eosin (**J**). Scale bar, 25 µm. Data are presented as Mean ± SEM. * *p* < 0.05 vs. Bim^fl/fl^ mice.

**Figure 4:**
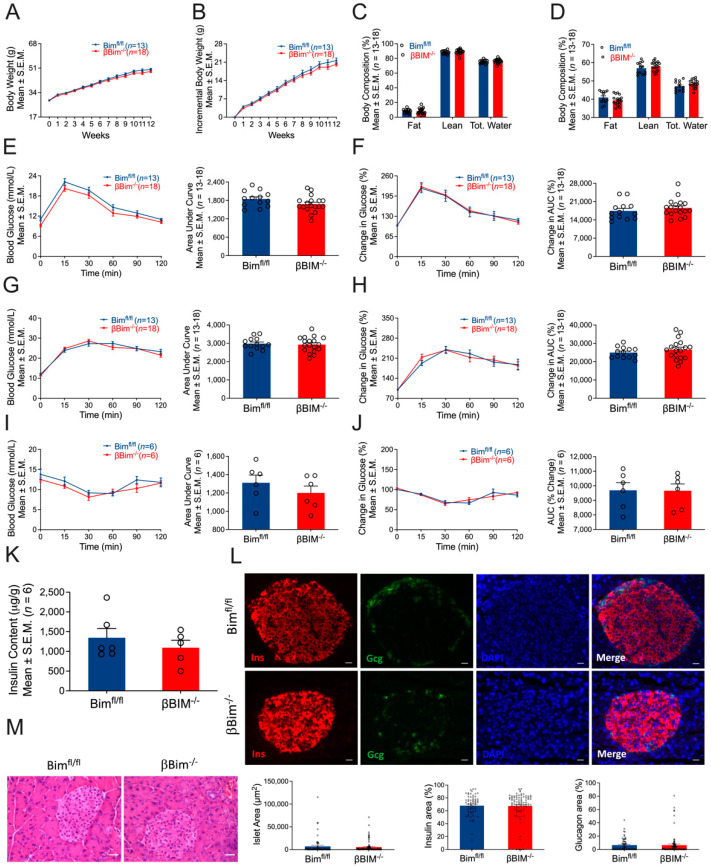
Effect of high-fat diet on glucose homeostasis of βBim^−/−^ mice. βBim^−/−^ mice were subjected to a high-fat diet regimen for 12 weeks. Body weight was measured weekly (**A**,**B**). Body composition (fat mass, lean mass, and total water) was measured before and after 12 weeks of high-fat diet feeding (**C**,**D**). Glucose tolerance tests measured before (**E**,**F**) and 12 weeks after high-fat diet feeding (**G**,**H**) did not show change in βBim^−/−^ mice compared to Bim^fl/fl^ mice. Similarly, an insulin tolerance test measured one week after the IPGTT did not show any difference between the two genotypes (**I**,**J**). Pancreatic insulin content was measured in an acid–ethanol extracted fraction using a commercial ELISA kit (**K**). Pancreatic sections were stained for insulin, glucagon, and DNA (**L**). **Bottom panel** (**L**) showing islet area, insulin, and glucagon positive cells (calculated as % of total islet area). Representative of 3 mice per genotype. Scale bar, 20 µm. Histology of mouse pancreas stained by Hematoxylin and Eosin (**M**). Scale bar, 25 µm. Data are presented as Mean ± SEM.

The Bcl-2 homology domain 3 (BH3)-only protein Bim is a critical mediator of cytokine-induced β-cell death and it has been shown that Bim is involved in β-cell apoptosis induced by β-cell Nova1 deficiency [18]. However, the β-cell-specific role of Bim in vivo remains elusive. Therefore, we generated a mouse model lacking Bim specifically in β-cells using a Cre-Lox approach similar to that used for βNova1^−/−^ mice. Bim deletion was confirmed in the β-cells of βBim^−/−^ mice (Figure 3A). Bim deficiency in the β-cells did not alter diabetes development induced by MLD-STZ, body weight or composition (Figure 3B–D and Figure S6A–H), and weekly blood glucose (Figure 3E; top and bottom panels). At the end of 6 weeks, IPGTT performed in βBim^−/−^ and littermate control mice did not show any change in glucose tolerance indicated by blood glucose levels (Figure 3F; top and bottom panels) and % change in blood glucose (Figure 3G; top and bottom panels) after glucose bolus injection during IPGTT. The insulin content measured in the pancreas of these mice was indistinguishable between the groups (Figure 3H). Immunofluorescence analysis of pancreatic sections showed that the percentage insulin area was slightly reduced (*p* = 0.03) in βBim^−/−^ mice (Figure 3I; top and bottom panels). However, H&E staining did not show any apparent changes between the two genotypes (Figure 3J). Collectively, these results show that Bim deletion in β-cells does not impair glucose homeostasis. Next, we subjected these mice to a 12-week HFD regimen to test the in vivo impact of Bim deletion in response to obesity-induced metabolic stress. After 12 weeks of the HFD, body weight (Figure 4A,B) and body composition (Figure 4C,D) were similar in βBim^−/−^ and littermate control mice. Before starting the HFD, the glucose tolerance test was unchanged between Bim^fl/fl^ and βBim^−/−^ mice (Figure 4E,F). The HFD feeding did not affect the blood glucose levels measured during IPGTT (Figure 4G,H) or ITT (Figure 4I,J), between Bim^fl/fl^ and βBim^−/−^ obese mice. Similarly, pancreatic insulin content (Figure 4K), immunofluorescence staining (Figure 4L), and histological analysis of H&E (Figure 4M) did not show any major change between high-fat diet fed Bim^fl/fl^ and βBim^−/−^ littermate mice. Taken together, these results show that Bim deletion in β-cells does not improve glucose homeostasis in obese or MLD-STZ-mediated diabetic mice. 

## 4. Discussion

The importance of alternative splicing in diabetes and pancreatic β-cell function is gaining interest. Previous findings have suggested an important role for NOVA1 in pancreatic β-cell function and survival including the following: (i) studies showing expression of NOVA1 in human pancreatic islets [15] and purified rat β-cells [34]; (ii) NOVA1 knockdown in vitro induced apoptosis (basal and cytokine-induced) in human islets impairs glucose-stimulated insulin secretion in INS-1E cells and regulates transcripts involved in exocytosis, apoptosis, insulin signaling, splicing, and transcription in FACS-purified rat β-cells [18]. Despite this convincing in vitro evidence, the in vivo function and physiological relevance of NOVA1 in β-cells remain unclear. Here, we determined the in vivo metabolic effect of β-cell-specific Nova1 deletion in mice to directly test the hypothesis that the loss of Nova1 in β-cells will adversely affect glucose homeostasis. Surprisingly, we did not detect any changes in glucose-stimulated insulin secretion in mice with a β-cell specific loss of Nova1. Conversely, βNova1^−/−^ mice islets demonstrated a trend towards increased—albeit not significant—insulin secretion under high glucose stimulation. Nova1 is known to be indispensable for neuronal survival as Nova1-null mice die postnatally due to neuronal apoptosis [35] and motor neuron dysfunction [36]. However, βNova1^−/−^ mice did not show any adverse phenotypes, suggesting that Nova1 may not be necessary for β-cell survival or function. Since we did not observe any spontaneous adverse phenotypes in βNova1^−/−^ mice, we reasoned that Nova1 may be necessary under pathological conditions. Therefore, we challenged Nova1 deficient β-cells with inflammatory stress (MLD-STZ) or moderate metabolic stress (insulin resistance) conditions. To address this question, we subjected these mice to two independent models of metabolic stress with impaired β-cell function. First, we used MLD-STZ-induced diabetes, which is a widely used model for studying β-cell inflammation and death. We injected STZ to Nova1^fl/fl^ and βNova1^−/−^ mice and followed them for 6 weeks. We did not see any changes in blood glucose, glucose tolerance (only a trend towards a reduction at later time points after glucose bolus), or pancreatic insulin content between the two genotypes. Second, it is established that a long-term high-fat diet enhances β-cell proliferation and mass [37]. Therefore, to test the impact of Nova1 loss in β-cells, we fed βNova1^−/−^ mice a high-fat diet. No difference in body composition and pancreatic levels was observed between the two genotypes. Surprisingly, βNova1^−/−^ mice exhibited a moderate improvement in HFD-induced impaired glucose tolerance compared to Nova1^fl/fl^ mice. Overall, from two metabolic stress mouse models, our data clearly argue against a β-cell-specific physiological/pathological role of Nova1. 

It has also been shown that Nova1 knockdown in INS-1E cells induces basal and cytokine-induced apoptosis and this cell death is mediated by the BH3-only protein Bim [18]. Both in vitro and in in vivo studies [38,39] have previously shown that Bim is downstream Nova1 and plays an important role in β-cell apoptosis in diabetes. Moreover, Bim activates apoptosis in several models of β-cell stress and high mRNA levels of Bim is detected in islets of T2D patients [21]. These findings collectively suggest that BIM plays a physiological metabolic role. However, the impact of β-cell-specific role of Bim in glucose homeostasis in vivo has not been previously studied. We generated a mouse model with β-cell-specific deletion of Bim and found indistinguishable parameters both under physiological condition and in response to inflammatory-mediated diabetes or HFD-induced obesity. Hence, our finding shows for the first time that the loss of Bim in β-cells is not sufficient to improve β-cell function and/or survival in vivo.

Thus, the question remains: why did the loss of Nova1-Bim in β-cells have no effect on glucose homeostasis in vivo? Several plausible reasons emerge that may explain the discrepancy between previous strong in vitro data supporting a beneficial role of Nova1 in β-cells and our in vivo findings in this study. First, though NOVA1 expression has been detected in human pancreatic islets [15] and purified rat β-cells [34], their expression pattern in the diseased context is largely unknown. Detailed characterization of pancreatic NOVA1 expression in diabetic patients or rodent models of diabetes would help to determine if altered NOVA1 expression is adaptive or maladaptive during diabetes. Second, it is possible that β-cells-specific Nova1 knockout induces a compensatory increase of alternative RBPs other than Nova2. This is not in line with the indispensable role of Nova1 in neurons. Global knockout mice of Nova1 die postnatally from a motor deficit associated with apoptotic death of spinal and brainstem neurons [35]. It is conceivable that deletion of both Nova1 and Nova2 expression is necessary to induce β-cell dysfunction in vivo. In line with this, a recent study using adipocyte-specific deletion of Nova1 and Nova2 has shown an additive effect, promoting adipose tissue thermogenesis and improving glycemia [23]. Finally, to our knowledge, the study by Villate O et al. is the only study that has used multiple in vitro models to show the importance of Nova1 in β-cell function and survival [18]. Therefore, it is important to consider that in vitro studies may provide molecular insights into cellular processes, but they do not often completely recapitulate the complexity of in vivo physio and pathophysiology, which makes it difficult to translate the relevance of the findings.

A limitation of our studies is that we focused our in vivo work on adult male mice, but the role of Nova1 and Bim in β-cell function should also be clarified in the development of obesity and diabetes at different ages and in females [40]. In addition, we have not performed mechanistic/gene sequencing studies to identify altered gene expression or post-transcriptional gene modifications by knocking out Nova1 or Bim in β-cells. In the present work, our main goal was to determine whether deficiency of Nova1 or Bim in β-cells has an in vivo metabolic impact and whether previous in vitro findings can be translated to an in vivo set up. In summary, our data provide evidence against a role for Nova1 and Bim in β-cell function. 

## 5. Conclusions

The loss of functional pancreatic β-cell mass is an important hallmark of both T1D and T2D. Our data from two independent mouse models provide evidence against a critical role for Nova1 and Bim in β-cells and do not support their modulation to improve β-cell survival. It is important to note, however, that our data do not completely rule out a potential contribution of NOVA1/BIM to β-cell dysfunction in human T1D or T2D.

## Data Availability

The data presented in this study are available within this article and supplementary figures. The data presented in this study are available on request from the corresponding author.

## Supplementary Materials

The following supporting information can be downloaded at: https://www.mdpi.com/article/10.3390/nu14183866/s1, Supplementary Figure S1: Nova2 expression is not affected in βNova1^−/−^ islets, Supplementary Figure S2: Change in body composition of βNova1^−/−^ mice after MLD-STZ administration, Supplementary Figure S3: Expression of insulin, glucagon and the macrophage marker CD68 in pancreas of MLD-STZ-treated βNOVA1^−/−^, βBim^−/−^ and control mice, Supplementary Figure S4: Transgenic expression of the Cre gene did not affect impaired glucose homeostasis induced by MLD-STZ, Supplementary Figure S5: Change in body composition of Ins1Cre mice after MLD-STZ administration, Supplementary Figure S6: Change in body composition of βBim^−/−^ mice after MLD-STZ administration.
