# Supplementary material for: Nova1 or Bim Deficiency in Pancreatic β-Cells Does Not Alter Multiple Low-Dose Streptozotocin-Induced Diabetes and Diet-Induced Obesity in Mice
Source: Nutrients. 2022 Sep 18;14(18):3866. doi: 10.3390/nu14183866 (PMC9500891; doi:10.3390/nu14183866)
Supplement: Supplementary file 1 [file nutrients-14-03866-s001.zip › nutrients-1535758-supplementary.pdf]

Supplemental Material.

Nova1 or Bim deficiency in pancreatic β-cells does not impact on multiple low-dose streptozotocin-induced diabetes and diet-induced obesity in mice.

Manoja K. Brahma, Peng Xiao, Madalina Popa, Javier Negueruela, Valerie Vandenbempt, Stéphane Demine, Alessandra K. Cardozo, Esteban N. Gurzov

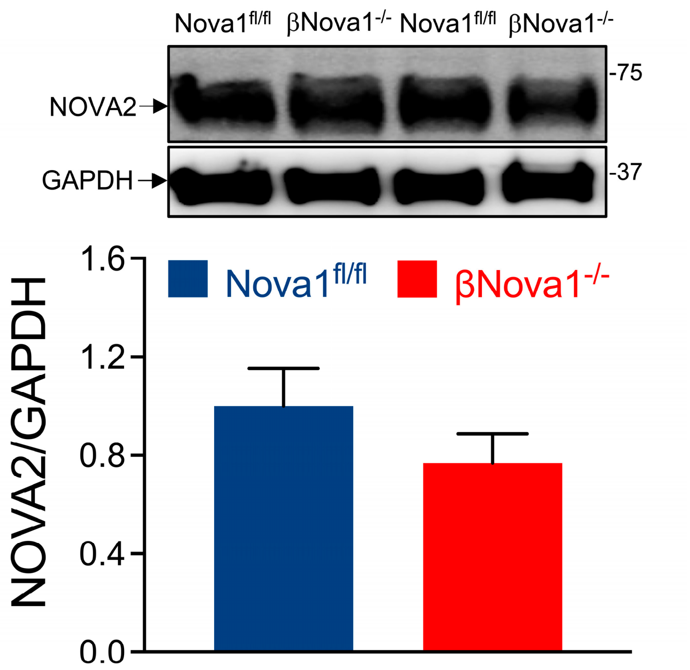

Figure S1. Nova2 expression is not affected in βNova1$^{-/-}$ islets. Nova1 was deleted in β-cells of mice using Cre-LoxP technology. Nova2 expression was assessed in isolated islets from Nova1$^{fl/fl}$ or βNova1$^{-/-}$ mice by immunoblotting using anti-NOVA2 antibody. n=3.

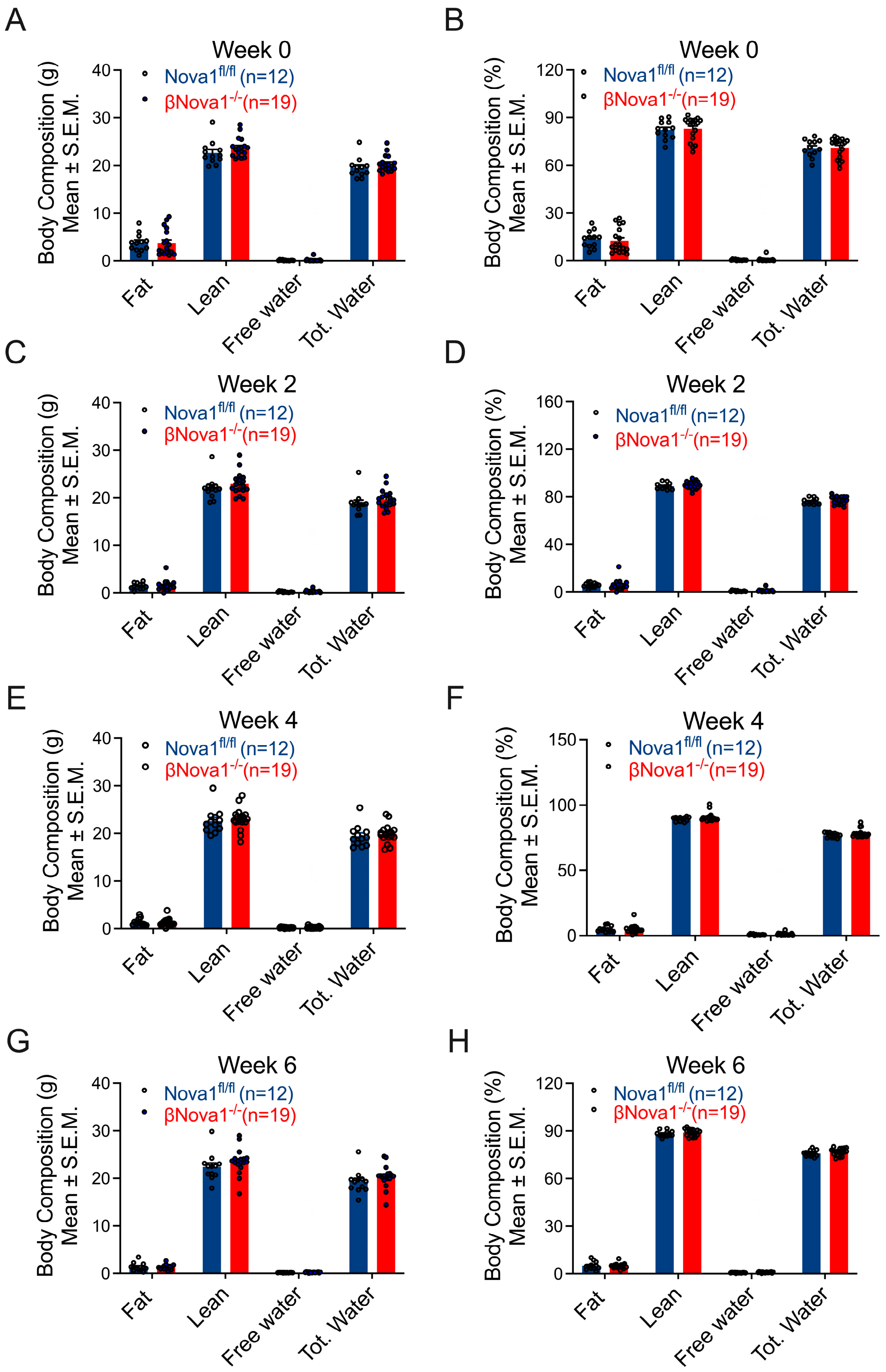

Figure S2. Change in body composition of βNova1$^{-/-}$ mice after streptozotocin administration. Body composition (fat mass, lean mass, free water and total water) was monitored before and after every 2 weeks of STZ administration. Left panel (**Fig. A**, **C**, **E**, and **G**) shows the body composition in grams and the right panel (**Fig. B**, **D**, **F**, and **H**) shows the body composition in % of the body weight.

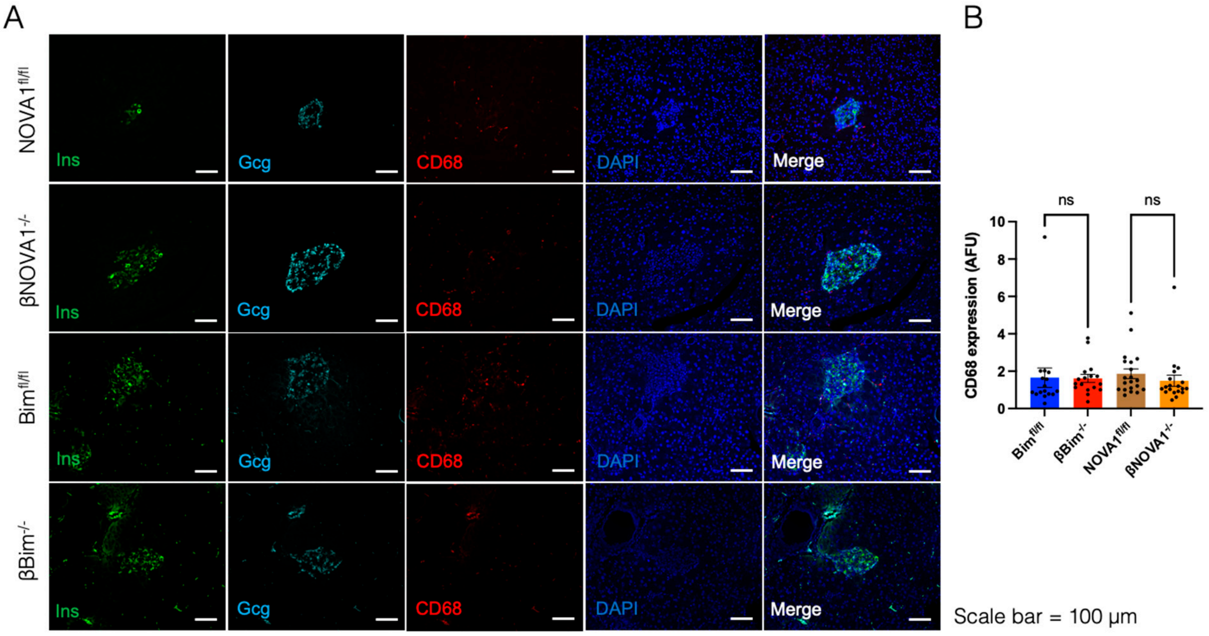

Figure S3. Expression of insulin (Ins), glucagon (Gcg) and the macrophage marker CD68 in pancreas of βNOVA1$^{-/-}$ and βBim$^{-/-}$ mice. Left panel (A) shows pancreatic sections stained for insulin, glucagon, CD68 and DNA (DAPI). Scale bar, 100 µm. Representative pictures of 3 mice per genotype. Right panel (B) shows the quantification of CD68 protein expression in the islet area (calculated as mean fluorescence intensity, expressed as arbitrary fluorescence units AFU)). Data are presented as mean ± SEM, each point corresponds to a different islet (n=16-20 islets, from 3 different mice per condition). Ns, non-significant differences.

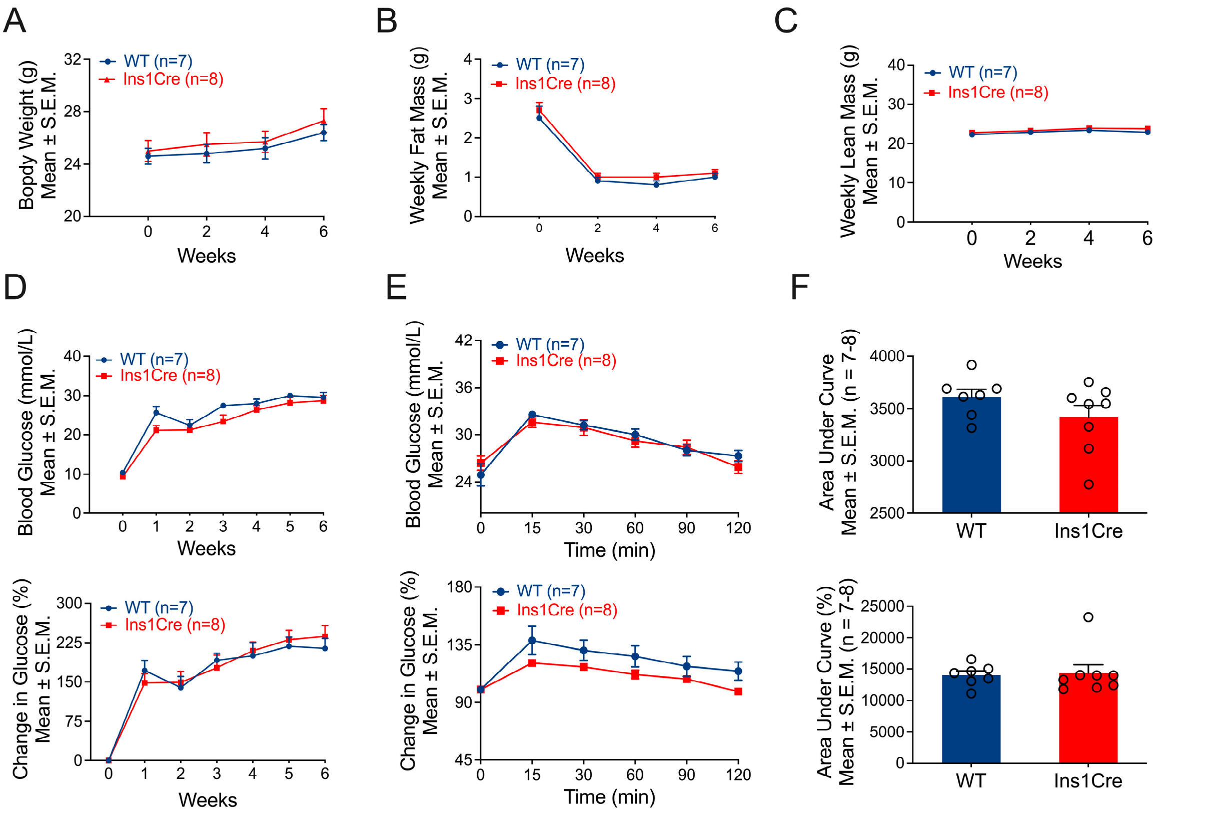

Figure S4. Transgenic expression of the Cre gene did not affect impaired glucose homeostasis induced by multiple low dose streptozotocin. Ins1Cre and wild type (WT) C57BL/6N mice were injected with 5-days of multiple low-dose of STZ and followed for 6 weeks. Body weight (**A**), body composition (**B** and **C**), and blood glucose (**D**; top and bottom panel) was monitored following STZ injection. Intraperitoneal glucose tolerance test was performed in fasted mice following 6 weeks of STZ injection. Blood glucose was measured expressed as mmol/L (**E**; top and bottom panel) or % change in blood glucose normalized to time 0 (**F**; top and bottom panel).

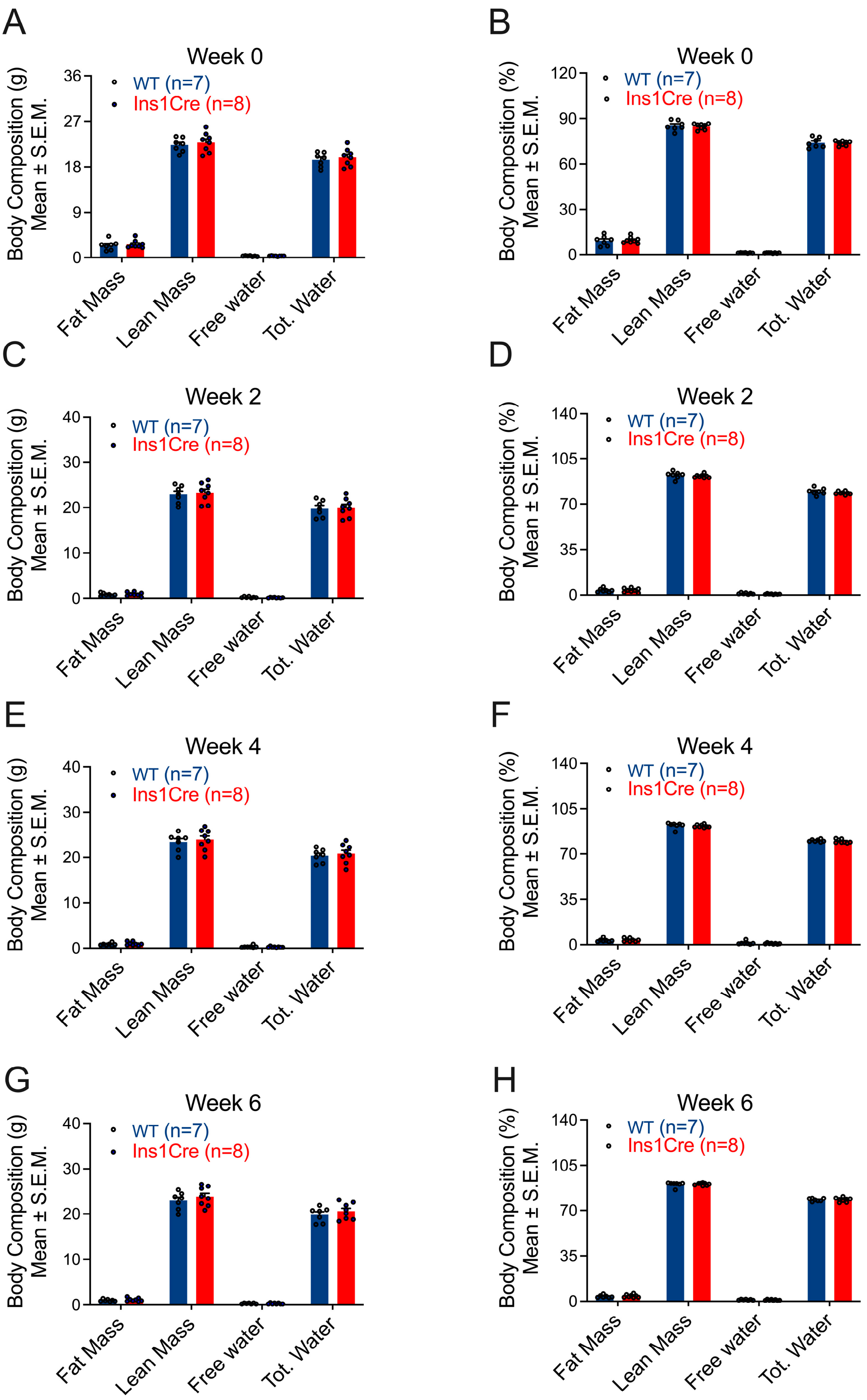

Figure S5. Change in body composition of Ins1Cre mice after streptozotocin administration. Body composition (fat mass, lean mass, free water and total water) was monitored before and after every 2 weeks of STZ administration. Left panel (**Fig. A**, **C**, **E**, and **G**) shows the body composition in grams and the right panel (**Fig. B**, **D**, **F**, and **H**) shows the body composition in % of the body weight.

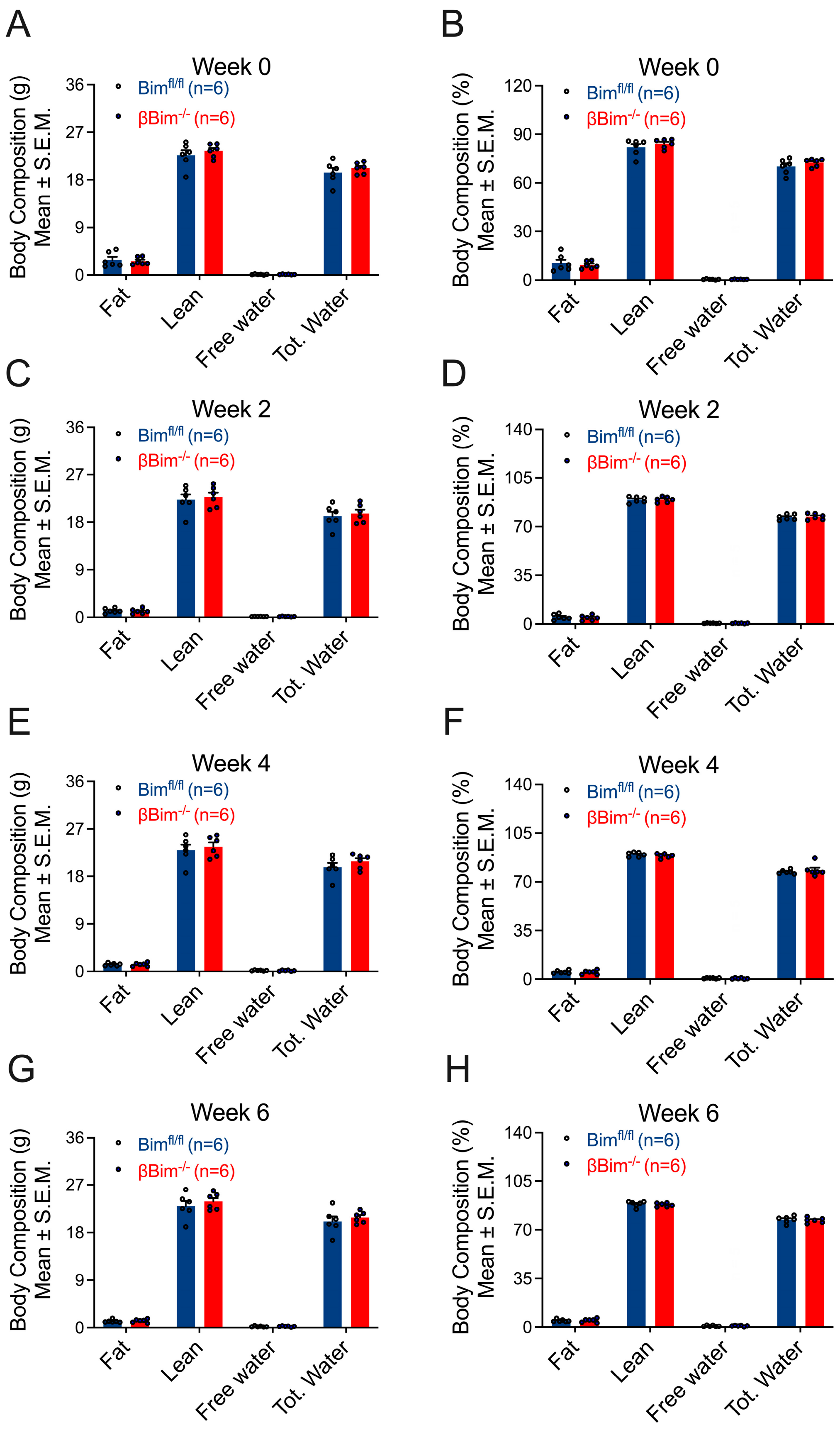

Figure S6. Change in body composition of βBim$^{-/-}$ mice after streptozotocin administration. Body composition (fat mass, lean mass, free water and total water) was monitored before and after every 2 weeks of STZ administration. Left panel (**Fig. A**, **C**, **E**, and **G**) shows the body composition in grams and the right panel (**Fig. B**, **D**, **F**, and **H**) shows the body composition in % of the body weight.
